# Supplementary material for: Factors associated with venous collaterals in patients with cerebral venous thrombosis
Source: PLoS One. 2024 Apr 16;19(4):e0302162. doi: 10.1371/journal.pone.0302162 (PMC11020378; doi:10.1371/journal.pone.0302162)
Supplement: Attachment — Submitted filename: Response to reviewers.docx [file pone.0302162.s002.docx]

**Response to reviewers’ comments**

Dear reviewers,

I would like to appreciate your sharp sight in this manuscript and valuable comments. Please find the response to reviewers’ comments in the table below:

| **Reviewer 1** | | |
| --- | --- | --- |
| **Comments** | **Response** | **Page and line of change** |
| In abstract, ensure that similar decimal places are used throughout (needs to be consistent) | Thank you for your comment.  We have thoroughly checked the number and decimal of all parameters and correct according to the statistic results shown in all tables. | Abstract  Page2 line26  Table 1 ,2 and 3. |
| Line 33 &ndash; the authors should be more specific, ie. In a cohort of patients with CVT, the frequency of collateral was &hellip; | We edited this section as the following:  In this cohort, the prevalence of venous collaterals was 25.3%. | Abstract  Page2 line33 |
| Line 38 &ndash; would rephrase this, many would not consider VST a type of stroke. | We edited this section as the following:  Cerebral venous thrombosis (CVT) is a potentially life-threatening veno-occlusive disease. | Introduction  Page3 line38 |
| Line 39 &ndash; this is redundant and out of place, please omit |  |  |
| Line 45 &ndash; should say that it can lead to hemorrhage. | We edited this section as the following:  the serious conditions including increased intracranial pressure and intracranial hemorrhage may occur. | Introduction  Page3 line44-45 |
| Line 57 &ndash; the authors need to clearly state why this study is being done, despite so many prior studies on VST and collaterals. What is unique about this study? What are the knowledge gaps and how does this study seek to fulfill the gap? | To emphasize these points, we edited this section as the following:  Venous collaterals arising from occluded sites are occasionally found in the radiographic studies of patients with CVT. However, it’s significance is not well established. Based on the current evidence, only 4 studies had objectively investigated the venous collateral in the different aspects. In terms of the clinical outcome prediction, these studies showed discordant results; either positive, negative or undetermined effect on the clinical outcome [1-3]. In the viewpoint of radiological outcome, a single study revealed the prediction of early recanalization [4].  Apart from the predictive role of venous collateral, several issues have never been mentioned. These gaps of knowledge include the factor contributing to venous collateral generation, the proper neuroimaging technic to evaluate venous collateral, the reliable classification for venous collateral grading and the correlation of the classification to either the clinical outcome and the complications. In this study, we aimed to identify the independent factors associated with venous collaterals at the diagnosis of CVT. In order to correct the drawback from the previous study; in regard of the non-uniform venous collateral grading, we apply the Qureshi classification in our study. | Introduction  Page3 line46-58 |
| Line 65 &ndash; why not CTV? | Although the CT venography of the brain is considered as a reliable method for CVT diagnosis, it is quite difficult to accurately identify the venous collateral due to the limited image resolution. Therefore, we did not set CTV in the inclusion criteria  We stated this issue in the method section as the following:  Although the CT venography of the brain is considered as a reliable method for CVT diagnosis, the visualization of venous collateral is problematic owing to the limited resolution of the CT venography. Therefore, the CT venography was not set in the inclusion criteria. | Method  Page 4, line 66-68 |
| Line 85 &ndash; needs reference | We added the reference in this point. In addition, the sequence of reference was rearranged in both texts and reference list.  International League Against Epilepsy (ILAE) 2017 Classification [5]) | Definition of terms  Page5 line88 |
| Line 156 &ndash; please include frequency of patients with collaterals as well | We edited this section as the following:  In this cohort, the venous collaterals were found in 20 patients (25.3%) according to the Qureshi classification [6]. | Characteristics of venous collaterals  Page10 line 162-163 |
| - Why was malignancy not included as a risk factor? | The malignancy was already included in the table 2 by using term “ cancer” | No change |
| - Was traumatic VST included? If not, the authors should clarify these are spontaneous | We edited this section as the following:  Inpatient records of the patients diagnosed with spontaneous CVT | Method  Page4 line69 |
| - Was is the interrater reliability of grading these collaterals? | Thank you for this valuable comments. We re-analysis the raw data and found that there were 12 discordant results. The interrater reliability calculated by percentage agreement was 84.8%.  We edited the method section and the result section as the following  1.The interrater reliability of venous collateral grading was calculated by using percentage agreement.  2.The venous collateral grading was independently assessed by 2 neuroradiologists. The interrater reliability calculated by percentage agreement was 84.8%. The 12 discordant venous collateral findings were concluded by consensus | Method [Data collection]  Page 4  Line78-79  Results  [Characteristics of venous collaterals]  Page 10  Line 160-162 |
| - Not using gold standard uniform imaging in all patients should also be listed as limitation. For example, it likely easier to see collateral on DSA vs MRI. | We edited this section as the following:  First, the neuroimaging technic in this cohort was not uniform which might provide discrepant findings between the different method. | Strengths, limitations, and suggestions  Page18 line302-303 |
|  |  |  |
| **Reviewer 2** | | |
| **Comments** | **Response** | **Page and line of change** |
| 1. Your study describes some differences between patients with venous collaterals and those without, although you also mention how both the presentation and clinical outcomes in these two groups are not significantly different. May you elaborate on the clinical significance of the factors you identified as different between your two groups? How do you foresee the findings from your study being applied by neuroradiologists and neurologists evaluating patients with suspected or confirmed CVT? | We suggested the clinical implication and edited this section as the following:  Our study had expanded the limited current knowledge of venous collaterals in CVT. We suggested the clinicians and neuroradiologists to meticulously searching for venous collaterals in particularly the CVT patient who had superior sagittal sinus thrombosis or hormonal exposure. However, our experience found a difficulty to identify venous collateral accurately from the CT venography due to it’s limited image resolution. We suggested to use contrast-enhanced MRI or MRV as they provide the better visualization of venous collateral. | Strengths, limitations, and suggestions  Page18 line310-315 |
| 2. You mention that the prevalence of venous collaterals in your population was much lower than that seen in previous studies, including a study that used the same classification system as your study (the Qureshi classification) but found a prevalence of 88% (versus 25% in your study). Are there specific differences between the characteristics of your populations that would be expected to account for such a major difference in prevalence, or how can we best reconcile these differences? | We also concerned this wide gap of the prevalence difference between the studies. We have tried our best to search for evidence or comparing the data. As the studies focusing the venous collateral are very limited in number, we hardly find the sufficient data to explain this point. The previous studies had not mentioned about the detail of demographic data and etiologies, but focus primarily on the radiographic findings.  We have postulated the ethnicity difference may play a role in these prevalence difference. Though we have tried our best to search for scientific evidence; in regard of genetic polymorphism, there is insufficient scientific data to support this idea.  We accepted that we could not find the better explanation for this finding. The reliable reasons in our opinion are the difference in the neuroimaging technic to visualize venous structure and the different in the venous collateral grading classification as mention the discussion. | No change |

We would like to appreciate the superb reviewers’ comments which were very helpful to sharpen this manuscript.

Sincerely Yours,

Pat Korathanakhun, M.D.

On behalf of all authors
